# Variation in the Correlation of G + C Composition with Synonymous Codon Usage Bias among Bacteria

**DOI:** 10.1155/2007/61374

**Published:** 2007-07-25

**Authors:** Haruo Suzuki, Rintaro Saito, Masaru Tomita

**Affiliations:** 1 Institute for Advanced Biosciences, Keio University, Yamagata 997-0017, Japan

## Abstract

G + C composition at the third codon position (GC3) is widely reported to be correlated with synonymous codon usage bias. However, no quantitative attempt has been made to compare the extent of this correlation among different genomes. Here, we applied Shannon entropy from information theory to measure the degree of GC3 bias and that of synonymous codon usage bias of each gene. The strength of the correlation of GC3 with synonymous codon usage bias, quantified by a correlation coefficient, varied widely among bacterial genomes, ranging from 0.07 to 0.95. Previous analyses suggesting that the relationship between GC3 and synonymous codon usage bias is independent of species are thus inconsistent with the more detailed analyses obtained here for individual species.

## 

[1,2,3,4,5,6,7,8,9,10,11,12,13,14,15,16,17,18,19,20,21,22,23]
